# Suspected limited mobility of a Middle Pleistocene woman from Southern Italy: strontium isotopes of a human deciduous tooth

**DOI:** 10.1038/s41598-017-09007-5

**Published:** 2017-08-17

**Authors:** Federico Lugli, Anna Cipriani, Julie Arnaud, Marta Arzarello, Carlo Peretto, Stefano Benazzi

**Affiliations:** 1 Department of Chemical and Geological Sciences, University of Modena and Reggio Emilia, Via Campi 103, 41125 Modena, Italy; 2 Lamont-Doherty Earth Observatory, Columbia University, Palisades, New York, 10964 USA; 3 Department of Humanities, Section of Prehistorical and Anthropological Sciences, University of Ferrara, C.so Ercole I d’Este 32, 44121 Ferrara, Italy; 4 Department of Cultural Heritage, University of Bologna, 48121 Ravenna, Italy; 5 Department of Human Evolution, Max Planck Institute for Evolutionary Anthropology, 04103 Leipzig, Germany

## Abstract

We present the Sr isotopic composition of enamel of the most ancient deciduous tooth ever discovered in Italy to assess human mobility in Middle Pleistocene. Reconstructing ancient mobility is crucial for understanding human strategy at exploiting temporally and spatially patchy resources, with most studies focusing on indirect evidences, ultimately affecting our interpretation on hominin territoriality and energetic costs invested by hominin groups. Here, we use the high spatial resolution and micro-destructivity options offered by the Laser Ablation Multi-Collector Inductively Coupled Plasma Mass Spectrometry technique, to determine the ^87^Sr/ ^86^Sr intra-tooth variability of a human deciduous incisor from the Middle Pleistocene layers of the Isernia La Pineta site (Italy). We compared these data with the Sr isotopic signature of local micro-mammals, the broadest home-range of the macro-mammals and with modern plant samples. Our study reveals that while macro-mammals have possibly migrated through the landscape for up to 50 km, the pregnant woman from Isernia was probably local, given that the isotopic ratio of the enamel falls within the local range and is comparable with the signature of the local plants in a radius of 10 km. This is the first case study of Sr isotopic composition determination in such ancient deciduous tooth.

## Introduction

Understanding how much mobility or sedentary has influenced human evolution is a major challenge in human past ecology. Mobility patterns of human groups have deep implications on their exploitation of the landscape resources, proving whether their adaptation strategies are more or less successful in relation to climatic changes.

Human mobility of ancient communities and the consequent interaction with the environment and other populations is often hard to define because of a lack of concrete data. This is especially true for the Middle Pleistocene, given the very low number of sites/findings and the lack of preserved materials. Commonly, the investigation of hominin past mobility has been achieved using different indirect evidences as, e.g., lower limb morphology^1^, raw material procurement^2^, diverse exploitation of faunal resources^3^ and study of the camp size^4^. However, these approaches give a patchy overview of the resource exploitation and environmental change adaption because they inform about specific hominins within the analyzed site. For example, it is not known whether the subsistence strategies observed in this site account for a short-term occupation of the area due to the depletion of local resources, and the consequent need to move to other regions, or whether the group undertook fewer residential moves to exploit more intensively the resources at hand. A detailed work about these mobility strategies has been done by Lewis R. Binford^5^ on recent hunter-gatherer groups. He differentiated two mobility strategies as follows: the residential mobility, where the so-called *foragers* practice frequent residential base camp moves to reach major resource patches, and the logistic mobility of the *collectors*, practicing fewer residential moves and logistical mobility from the base camp to exploit spatially and temporally patchy resources^5^.

During the last decade, strontium isotope (^87^Sr/^86^Sr) analyses have been exploited in the study of human past mobility and landscape exploitation because of the special relationship between the Sr isotopic fingerprint stored in human bones and teeth and the living location of the individual (e.g. refs 6–10). For example, Copeland *et al*.^9^ studied the Sr isotopic composition of permanent enamel in early hominins (~2 million years ago) from South Africa (Sterkfontein and Swartktrans) showing that only about 30% of the analyzed individuals (*Paranthropus robustus* and *Australopithecus africanus*) were non-consistent with the local dolomite Sr isotope composition and, therefore, were considered non-local. Their comparison between the proportion of non-local individuals between small (probably female) and large hominins (probably male) shows that at least 50% of the female were non-local in contrast with the 11% of non-local male, suggesting a different landscape use between adult males and females^9^. In contrast, Balter *et al*.^10^ Sr isotope analyses of three different hominin groups (*P*. *robustus*, *A*. *africanus* and early *Homo*) from the same region, including also browser and grazers, revealed that the groups show similar home range areas, indistinguishable from the local fauna.

Richard *et al*.^11^ defined a supply region of about 20 km for a *Homo neanderthalensis* from Greece (Lakonis site). The enamel of this individual presented a quite large range of Sr isotope ratios (on average 0.7107 ± 0.0010; recalculated from the data of Richard *et al*.). The more radiogenic values observed in the enamel, compared with lower local isotope ratios, and the great variability within the tooth led the authors to hypothesize that the individual spent part of its childhood far away from the site/region in which it was found, at least 20 km, and that this Neanderthal group practiced residential mobility.

Recently, Willmes *et al*.^12^ reported three Sr isotope ratios of a *Homo neanderthalensis* enamel (unerupted molar) from Payre (France) ranging from 0.71084 to 0.70902 suggesting a high mobility of the studied individual.

Despite the great potential of the Sr isotope method application to mobility studies, there has been no attempt to use it in Middle Pleistocene specimens. To our knowledge, mobility patterns in Middle Pleistocene hominins have only been inferred by observations of raw material exploitation for lithic artefacts, from butchery testimonies and from environmental data^3^. Overall, these evidences suggest that the presence of a rich and diversified fauna coupled to a less cold environment in central and southern Italy compared to northern regions, may have led to the local adaptation of hominin groups^3^.

In this paper, we analyze the strontium isotopic composition of a deciduous human incisor from the Middle Pleistocene site of Isernia La Pineta (Molise, Italy) through a micro-destructive technique, namely LA–MC–ICP–MS (Laser Ablation Multi-Collector Inductively Coupled Plasma Mass Spectrometry). Given that deciduous teeth form within the uterus of pregnant women, the aim of this work is try to determine the mobility pattern of the mother of the individual, and ultimately the role that (pregnant) women may have played within archaic human groups.

## Results

**Figure 1:**
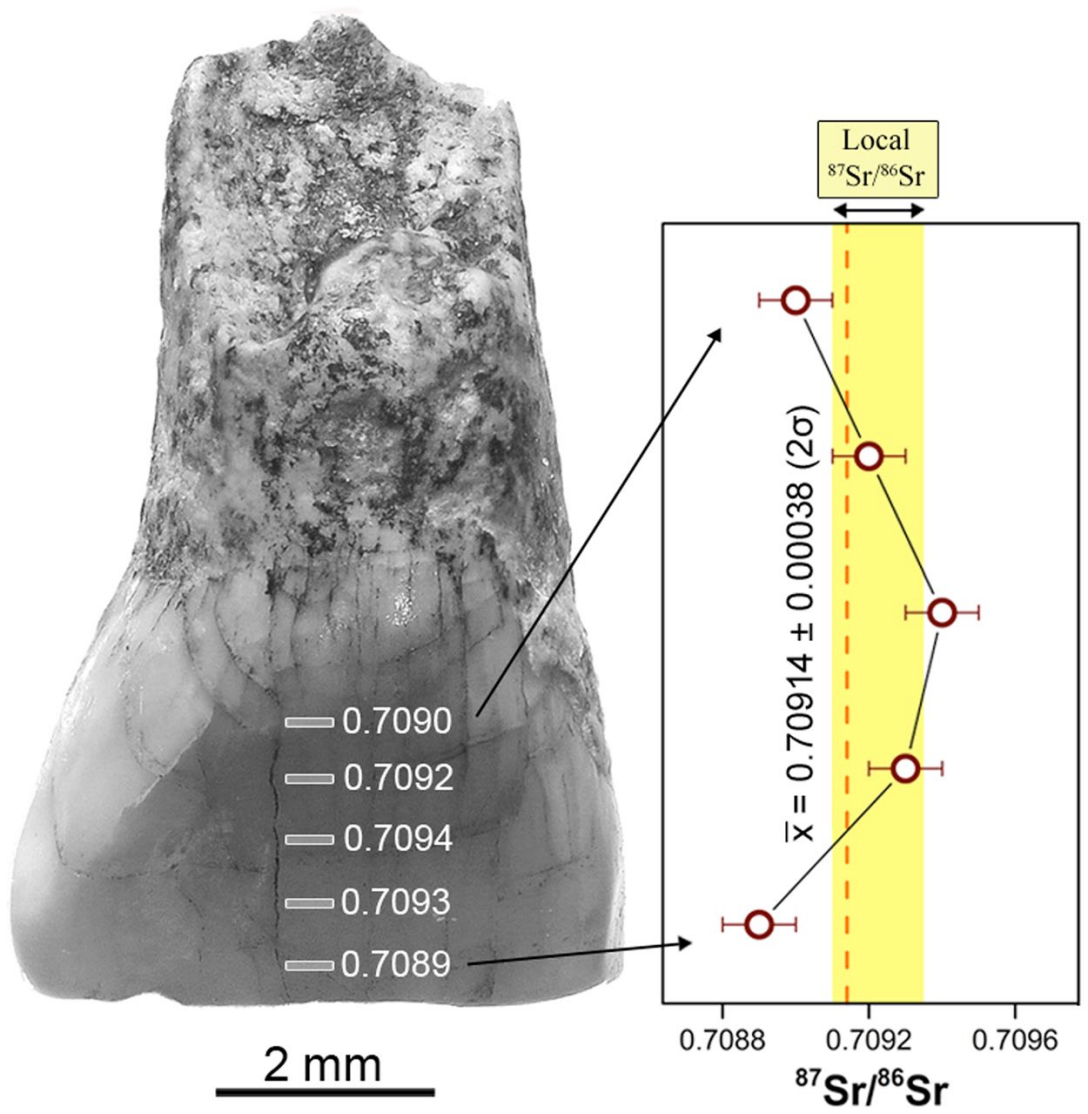
Human deciduous incisor IS42 from Isernia La Pineta and *in situ* Sr isotope results.

The strontium isotope ratios of the human tooth and animal teeth are summarized in Table 1 and Table S1. Analyses by LA–MC–ICP–MS of the human tooth IS42 (Fig. 1), yielded an average ^87^Sr/^86^Sr ratio of 0.70914 ± 0.00038 (2σ; 5 ablation lines) and a Sr concentration of 87 ± 20 ppm.

**Table 1:** LA–MC–ICPMS data of IS42 (deciduous human tooth) enamel.

**Sample**	^**87**^ **Sr/** ^**86**^ **Sr**	**Sr (ppm)**
IS42a	0.70896	70
IS42b	0.70925	83
IS42c	0.70936	90
IS42d	0.70925	97
IS42e	0.70887	92
**Average ± 2σ**	**0.70914 ± 0.00038**	**87 ± 20**

All ^87^Sr/^86^Sr 2SE (in-run error) are ≈ 0.00010. Sr concentration was estimated through linear interpolation from known standard materials during the analytical session.

Several rodent teeth were analyzed by dissolution MC–ICPMS and their isotopic composition varies between 0.70915 and 0.70938 (average 0.70924 ± 0.00013; 2σ; n = 16), whilst their Sr concentration varies between 166 and 392 ppm. Concerning the macro-fauna, we have analyzed by dissolution MC–ICP–MS seven bison teeth. Their ^87^Sr/^86^Sr ratio ranges between 0.70927 and 0.70954 (average 0.70938 ± 0.00018; 2σ; n = 7), while their Sr concentration between 94 and 649 ppm. Furthermore, we have analyzed nine rhino teeth (two specimens represent different portions of the same tooth) by dissolution MC–ICPMS. These specimens show a ^87^Sr/^86^Sr ratio ranging from 0.70941 and 0.70979 (average 0.70958 ± 0.00030; 2σ; n = 9) and a Sr concentration ranging from 209 to 514 ppm.

No significant statistical difference is observed between the ^87^Sr/^86^Sr ratios of the human tooth and the rodent samples (*p* = 0.7726; two-tailed Mann-Withney), suggesting that the isotopic signal of IS42 falls within the local variability. Contrariwise, both bisons (*p* = 0.0012; two-tailed Mann-Withney) and rhinos (*p* < 0.0001; two-tailed Mann-Withney) show a Sr isotope ratio statistically different from the local ^87^Sr/^86^Sr ratio (~0.7096 vs. ~0.7092). The difference between the animal average ^87^Sr/^86^Sr ratio and the local average ^87^Sr/^86^Sr ratio (∆_local_) is 0.00014 for the bison and 0.00034 for the rhino.

The baseline of the bioavailable strontium of the surrounding area has been inferred from modern plant specimens and their isotopic ratios range from 0.7096 to 0.7083 (see Table S2). Plant samples have been divided in two groups based on the distance from Isernia La Pineta (Table S3). The group of samples collected at less than 15 km from the site shows a ^87^Sr/^86^Sr average value of 0.7087 ± 0.0005 (2σ), whilst the group of samples at more than 15 km shows an average value of 0.7088 ± 0.0011 (2σ). One archaeological rodent tooth from Grotta Reali (c.a. 20 km from Isernia) has a Sr isotope ratio of 0.70884. In addition, Sr isotopic data of lavas from Conticelli *et al*.^13^ have been reported as the local value for the area of Roccamonfina at about 35–40 km southward, with the highest radiogenic values of about 0.7100.

## Discussion

The average ^87^Sr/^86^Sr ratio obtained for the human tooth IS42 (0.70914) fits within the local home isotopic range as defined by the 2σ and the average of the rodent teeth (0.70924 ± 0.00013).

**Figure 2:**
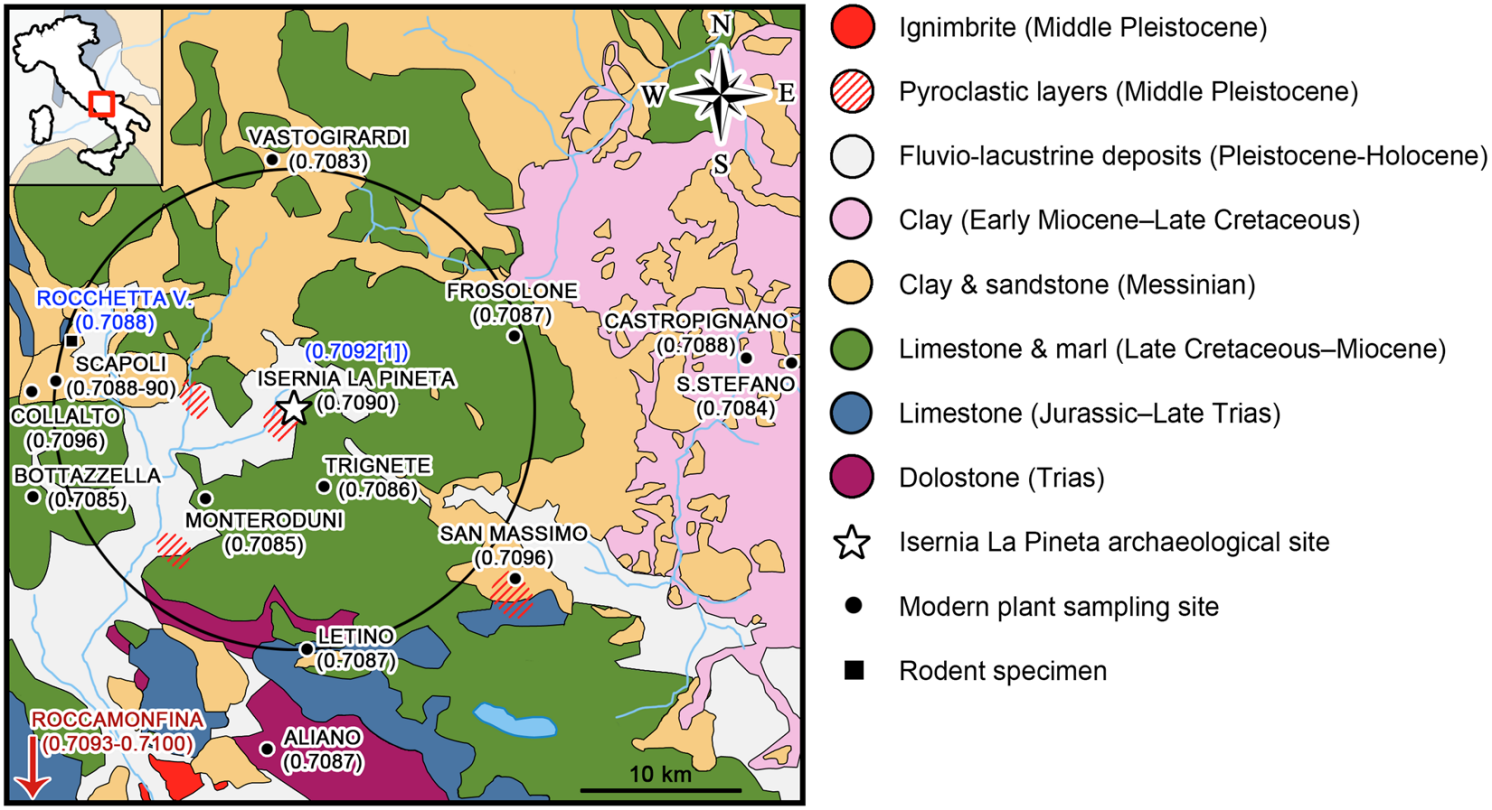
Simplified geological map of the Isernia area^39^ with location of the sampling sites. Black numbers in parenthesis are ^87^Sr/^86^Sr ratios of modern plant specimens; blue ^87^Sr/^86^Sr ratios are rodent tooth specimens (data from Rocchetta a Volturno and local rodents from Isernia La Pineta), red ^87^Sr/^86^Sr ratios are Roccamonfina (~10 km southward) leucites^13^. Pyroclastic layers (oblique red lines) are documented in several localities by^18^. Black circle represents an area of 15 km surrounding Isernia La Pineta. Drawing by Federico Lugli (INKSCAPE 0.92, inkscape.org).

The Isernia La Pineta site (Fig. 2) is locally dominated by Holocene and Pleistocene fluvial-lacustrine deposits, while the surrounding area (about 30 km away) consists mainly of carbonate and terrigenous deposits formed between the Mesozoic and the Cenozoic^14^. Unfortunately, no isotope data are available in the literature from waters, sediments or carbonate rocks of the area. However, we can indirectly infer them from the seawater ^87^Sr/^86^Sr curve of McArthur *et al*.^15^. The McArthur curve derives from measurements of ^87^Sr/^86^Sr ratio of biostratigraphically well-dated marine sediments and fossils (e.g. shells, sedimentary rocks, evaporite rocks) and is broadly exploited in dating of marine strata, through the so-called Strontium Isotope Stratigraphy methodology^15^. Therefore, the Sr isotopic signature of sedimentary rocks formed during the time-stretch of geologic interest to the Isernia La Pineta site should lie somewhere between 0.70730 (140 Ma) and modern seawater 0.70917^15^. Animal bones from the Gargano promontory^16^, about 200 km east of Isernia, with outcropping rocks from the Trias to the Miocene, show an ^87^Sr/^86^Sr range from 0.70817 (caprovine from Grotta Scaloria, Neolithic site) to 0.70863 (caprovine from La Torretta/Poggio Imperiale, Neolithic site). These isotopic values fit the Sr isotope ratios predicted by the McArthur curve for the age range of the sedimentary rocks outcropping at the two sites. Therefore, these animals have lived in a substratum with Oligocene-Miocene rocks and/or have ingested food with such a provenance, which means that they lived locally in the Gargano area. One shell from Poggio Imperiale, a locality close to the sea, shows, as expected, a Sr isotope ratio of 0.70909, close to modern seawater^16^. A rodent tooth from Grotta Reali, a Mousterian settlement located about 20 km west of Isernia La Pineta, has an ^87^Sr/^86^Sr ratio of 0.70884. This area is characterized by carbonate (Jurassic-Lower Miocene) and flysch (Upper Miocene) sedimentary sequences^16^ with a predicted Sr isotope range between ~0.7070 and 0.7090^15^. Again, the rodent tooth Sr isotopic composition fits within this range and corresponds to a Tortonian age, characteristic of the flysch deposits. The rodents of Grotta Reali and the caprovine of La Torretta/Poggio Imperiale confirm the fact that the local Sr isotopic composition fits the local geological background.

Given the small home range of rodents that reflects the local geology, as also extensively reported in literature (e.g. refs 8, 9, 17), we should expect the Sr isotope ratios of the local geology of Isernia La Pineta inferred from the McArthur curve (and therefore ranging between 0.70730 and modern seawater 0.70917) to reflect those of the rodents. Instead, we found that rodents from Isernia La Pineta have a mean ^87^Sr/^86^Sr ratio of 0.70924 ± 0.00013, slightly more radiogenic than expected. Peretto *et al*.^14^ have shown that pyroclastic deposits are intercalated within the sedimentary layers of the site, although this is not reported in the official geological maps of the area available in the literature. Moreover, several pyroclastic layers (Fig. 2) have also been documented by^18^. Therefore, volcanic material could have contaminated the Sr isotopic composition of local water and soil. The closest volcano to the Isernia site is the extinct Roccamonfina volcano, at about 40 km to the southwest, which was active between 630 and 50 ka. Its lavas have petrologic characteristic consistent with a convergent plate boundary with the low K series lavas showing ^87^Sr/^86^Sr ratios ranging from 0.7064 to 0.7083 and the high K series lavas from 0.7085 to 0.7100^19, 20^. The oldest volcanic activity (630 ka) was dominated by leucite-bearing lava flows, with an average ^87^Sr/^86^Sr ratio of 0.70969 ± 0.00059 (2σ; calculated from^13^). The Roccamonfina lavas are also characterized by very high Sr concentration, ranging from 774 to 1188 ppm in the low K series, from 1348 to 2358 ppm in the high K series and from 1670 to 2190 (average 1875 ± 388 ppm) in the oldest leucite-lavas^13, 19, 20^. Another nearby volcano showing high ^87^Sr/^86^Sr ratios is the Colli Albano volcano, located at about 150 km to the northeast of Isernia. Its lavas have ^87^Sr/^86^Sr ratios higher than 0.7100^21, 22^ and Sr concentrations up to ~2200 ppm. The highly radiogenic values of these volcanic rocks may explain the slightly higher ratios observed in the rodent teeth with respect to the local geology. Given the high Sr concentration of the volcanic rocks, a small amount of volcanic material can strongly contribute to the contaminations of the soils and therefore transfer the radiogenic isotopic signature to the local fauna.

The rhinos teeth of Isernia La Pineta have the highest ^87^Sr/^86^Sr ratios and isotopic variability of the analyzed fauna (2σ = 0.00030). Their relative Sr concentration (1/Sr) shows a strong negative correlation with their Sr isotope ratios (*n* = 9; *r* ^2^ = 0.64; *p* = 0.001), indicating that the highest-Sr reservoir has also the more radiogenic ^87^Sr/^86^Sr ratios (Fig. S1). The radiogenic Sr isotopic composition of the rhinoceros can be explained by the intake of water and food “contaminated” with the ^87^Sr/^86^Sr volcanic signature of the nearby Roccamonfina volcano. Recently, Lugli *et al*.^23^ detected cyclic variations in the enamel Sr isotopic composition of two rhinos teeth of Isernia La Pineta by *in situ* LA-ICP-MS high resolution investigations, with values ranging from 0.70937 to 0.70997. These variations have been correlated to the tooth growth features, suggesting that large mammals may have seasonally migrated southward in search of food, being attracted by the more fertile ground offered by the volcanic rocks of Roccamonfina^23^. Although farther away, even the Colli Albani area could have been reached by mammals during seasonal migration, as showed by the high ^87^Sr/^86^Sr ratios and Sr concentration of the lavas^22^.

Bisons have lower Sr isotope ratios and smaller variability than rhinos but their values are still higher than the human and rodents and are certainly also correlated to a larger or different area of supply that most likely reached the volcanic grounds.

The Sr isotopic values of the Isernia human tooth do not reflect a strong contamination by the volcanic radiogenic isotope signature of Roccamonfina (or Colli Albani).

**Figure 3:**
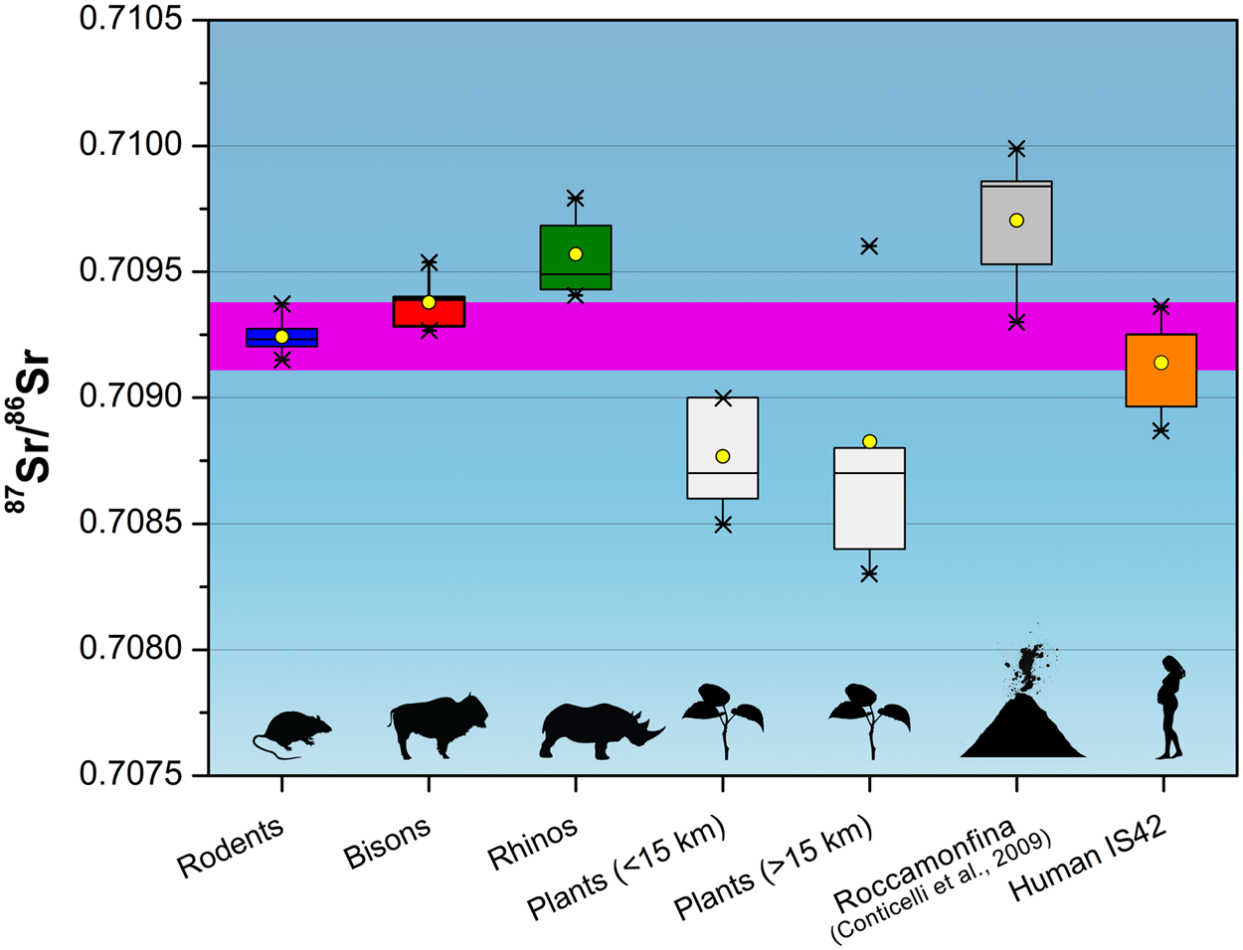
Strontium isotope data for human, fauna and modern samples of the Isernia La Pineta site and surroundings. The magenta area is the 2σ of the rodent teeth Sr isotope composition as the local ^87^Sr/^86^Sr reference value. Drawing by Federico Lugli (INKSCAPE 0.92, inkscape.org).

This suggests either a smaller supply area for the Isernia hominin compared to the large mammals of the site and/or a supply area not contaminated by volcanic deposits. More detailed inferences can be deduced from the isotope data of the local modern plants as a proxy for the bioavailable strontium. Considering together the local rodents (c.a. 1–2 km) and the within-15 km bioavailable strontium variability, the isotopic values observed in the human tooth fit within this range. The distant (>15 km) plants Sr isotope ratios and the Roccamonfina lava data are highly variable, and this variability seems not reflected in the human tooth (Fig. 3). The interpretations of the Sr homogeneous isotopic composition signal observed in this human tooth can be diverse. First, given that Central-Southern Italy is characterized by relatively young homogeneous limestone sediments^24^, even large human movements can be isotopically invisible if occurring on the same type of rock. Second, our knowledge on the Sr isotope composition of deciduous teeth in general is still far from being completely understood. In fact, it is not clear whether enamel tissue retains specific time-resolved information or if it is homogeneous because of the buffering of the Sr reservoirs of the mother bone (see Supplementary Information; refs 25, 26). Third, the low variability of the isotopic signal can be simply the result of a very limited supply area. The limits of the LA technique and the type of samples do not allow us to unequivocally choose between one of the hypotheses. Nevertheless, given that our data show macro-mammals moving through the landscape back and forth from marine sedimentary soils to volcanic soils, our preferred explanation for the local Sr isotopic composition of the human deciduous tooth of Isernia lies with a limited supply area and a suspected limited mobility of a pregnant woman in the Middle Pleistocene because of group strategy.

Human occupation at the Isernia La Pineta site occurred at the transition between the Marine Isotope Stage 15 (MIS15) and MIS14^14^. According to several climate and orbital proxies, since 700 kyr (mid-Pleistocene transition) a shift occurred from 41 kyr climatic cycles to strong 100 kyr cycles. The increase in amplitude in glacial-interglacial cycles caused an increase in the severity of glaciations. The first of these large-amplitude deglacial episodes is Termination VII (Marine Isotope Stage16–15 boundary), when global climate transitioned from the first very cold, extended glacial episode (MIS 16), to a relatively warm interglacial (MIS 15). In many sedimentary records, MIS 14 stands out as a short and much warmer glacial period than other glacial epochs of the last 800 kyr. It has been proposed that the extra-long duration of interglacial/mild stadial climates during MIS 15−13 may have provided favorable conditions over 100 kyr for the dispersal of African hominins into the Eurasia region during the Middle Pleistocene, leading to sustainable occupation^27^. In this climatic context, the human group of Isernia La Pineta, as most of the contemporary ones, seems to be not very mobile, probably exploiting local resources, a strategy likely similar to the modern collectors^3, 5^.

The suspected limited mobility suggested by strontium isotopes is in agreement with technological choices and hunting strategies^28^, which also attest for an evolutionary innovation in terms of mental templates. In fact, it seems that technical criteria employed in small débitage are not opportunistic and unstructured as previously thought, but reflect a well-established production method^14, 29–31^. Moreover, raw materials were collected by Middle Pleistocene *Homo heidelbergensis* mainly in fairly narrow areas and, seemingly, no large displacements were made for this purpose^2, 28, 30, 32^.

In terms of group strategy, the likely limited mobility of the pregnant women/mothers could also have had a huge impact on their role within the society and the gendered division of labor^33^. In recent hunter-gatherer communities, the general form in which the labor is subdivided can be summarized as men hunt and search for exotic resources, while women and children gather and exploit local resources. Moreover, women generally stay at camp to care for children, seldom following the men during long travels^33^. However, it seems that the gendered division of labor evolutionary appears with Upper Palaeolithic humans and was not exploited, for example, by Neanderthals^33^. Thus, the suspected reduced mobility of the Middle Pleistocene pregnant woman of Isernia could be in agreement with the division of labor observed in modern hunter-gatherer groups, a behavior risen perhaps earlier than we thought in human evolution.

The work of Wall-Scheffler and Myers^34^ also suggests that pregnant females of hominin groups might have changed the travel speed of the entire group and its mobility pattern. In particular, the persistent loads during pregnancy and child carrying slow the optimal walking speed of women and raise the energetic cost to walk with the group. Murray *et al*.^35^ state that *Pan troglodytes* pregnant females spent less time traveling than other females, reducing their physical activity, in order to store energy and fat for the forthcoming lactation. This strategy, as the authors say, is common in both humans and nonhuman primates, which can accumulate considerable fat reserves.

Despite the limited possibility of comparison with other human data of the same period, the tooth from Isernia shows the lowest Sr isotope internal variability yet presented in the literature in ancient hominins. Few studies on other species suggest that early hominins^9^ and Neanderthals^11, 12^ were characterized by larger mobility patterns, likely related to an adaptation strategy similar to modern foragers.

## Conclusion

In this paper, we present the first direct Sr isotope composition of a deciduous Middle Pleistocene (c.a. 570 ka) hominin tooth enamel. The ^87^Sr/^86^Sr ratios of this tooth are indistinguishable from the Sr isotope ratios of local rodents, possibly suggesting a local living location for the mother of the Isernia child. A limited mobility pattern hypothesis, similar to the logistical mobility of the modern hunter-gatherer collectors, is cautiously put forward, even if the data presented here are definitely not unambiguous. We cannot exclude mobility over geologically similar sedimentary rocks along the central portion of the Apennine chain. However, the comparison with modern plant specimens reveals the likely exploitation of local resources. Contrariwise, macro-fauna, namely rhinoceros and bisons, shows more radiogenic Sr isotope ratios, probably due to contamination with volcanic soils and likely related to seasonal migrations in the south-west direction, toward the Roccamonfina volcano area.

## Materials and Methods

### Laser Ablation MC–ICP–MS

The ^87^Sr/^86^Sr ratio of the IS42 human tooth was quantified by LA–MC–ICPMS technique on enamel micro-sampling carried out on the external surface of the tooth. We sampled 5 portions (linear transects) of the external enamel surface on the lingual side, following a cervical-incisal direction. Sr data were acquired with a double focusing MC–ICPMS Neptune™ (Thermo Fisher Scientific), coupled to a 213 nm Nd:YAG laser ablation system (New Wave Research^™^), housed at the Centro Interdipartimentale Grandi Strumenti (CIGS) of the University of Modena and Reggio Emilia, following the protocol described in Lugli *et al*.^23^. Laser parameters used during the analysis are those reported in Lugli *et al*.^23^. In order to maximize the ion beam size stability, we chose to use dynamic linear ablation (500 × 100 µm), which provides more precise results compared to static spot ablation^36^. To detect all masses required for the analysis (^88^Sr, ^87^Sr, 86.5, ^86^Sr, ^85^Rb, 85.5, ^84^Sr, ^83^Kr, ^82^Kr), nine Faraday detectors were employed. A 60 s background signal for each mass was collected before the analysis to correct for Kr interferences. ^83^Kr background signal was always lower than 1 mV. Signals on mass ^88^Sr of the IS42 sample ranged from 1.1 to 1.5 V. Instrument was tuned for low oxide production, constantly monitoring the ^87^Sr/^86^Sr ratio of reference materials. Moreover, before analysis, the ^83^Kr/^82^Kr ratio in the background was kept approximately equal to 1. Laser data were acquired in single blocks of 200 cycles, with an integration time of 0.5 s. To correct for the ^40^Ca^13^P^16^O formation we used our set of in-house bioapatite standards^23, 37^. The analysis of our human tooth in-house reference material (ROCht42) yielded an average (*n* = 10) LA ^87^Sr/^86^Sr ratio of 0.70865 ± 0.00040 (2σ) and an accuracy of 0.005% (16 ppm) compared with the previously obtained dissolution MC–ICP–MS value of 0.70864 ± 0.00001 (2se).

### Dissolution MC–ICP–MS

Several animal enamel samples (n = 32), including Microtinae indet. (n = 16), *Bison schoetensacki* (n = 7) and *Stephanorhinus hundsheimensis* (n = 9), were collected from the same archaeological level of the human tooth and from the two adjacent layers (immediately above and below; Table S1).

Modern plant specimens (n = 13) were collected in the area surrounding Isernia La Pineta, in a radius of about 30 km. Each specimen represents a pool of different plants (arboreal and herbaceous), collected in natural areas away from roads and cultivated fields, reflecting the average bioavailable strontium of the area. Before Sr extraction, plant samples were ashed at 500 °C^9^. One modern snail was also sampled from one of the modern plant site (Scapoli, see Table S2). Moreover, one archaeological rodent tooth from Grotta Reali (c.a. 20 km far from Isernia) was analysed.

Animal enamel samples, modern plant specimens and snail were analyzed after chemical dissolution and chromatographic separation of Sr. Samples preparation follows the protocol presented in Lugli *et al*.^23^. 5–10 mg of enamel and about 15 mg of plant ashes were digested in 1 ml of 14 N HNO_3_ and re-dissolved in 3 N HNO_3_ after evaporation. The Sr separation uses columns with a 300 µl volume filled with Eichrom Sr spec–resin. After several washes with MilliQ^®^ water and the conditioning of the resin with 3 N HNO_3_, samples were loaded into the columns and washed repeatedly with 3 N HNO_3_. Strontium was eluted with several reservoirs of MilliQ^®^ water. The whole procedure was conducted in a clean lab, with a Sr blank typically lower than 100 pg, at the Department of Chemical and Geological Sciences of the University of Modena and Reggio Emilia. Sr isotope ratios were determined by means of a MC–ICP–MS Neptune, housed at the CIGS (UNIMORE). A [blank/standard/blank/sample/blank] bracketing sequence was adopted to monitor any instrumental drift. Data were normalized through exponential law to a ^88^Sr/^86^Sr ratio of 8.375209^23^. During this work, the standard yielded a ^87^Sr/^86^Sr ratio of 0.71025 ± 0.00002 (2σ; n = 33). The Sr ratios were corrected for instrumental bias to the NBS–987 value of 0.71026 ± 0.00002 reported by^38^.

## Electronic supplementary material

Supplementary Information
