# Supplementary material for: Suspected limited mobility of a Middle Pleistocene woman from Southern Italy: strontium isotopes of a human deciduous tooth
Source: Sci Rep. 2017 Aug 17;7:8615. doi: 10.1038/s41598-017-09007-5 (PMC5561174; doi:10.1038/s41598-017-09007-5)
Supplement: Supplementary file 1 — Supplementary Information [file 41598_2017_9007_MOESM1_ESM.pdf]

Supplementary Information for:

Suspected limited mobility of a Middle Pleistocene woman from Southern Italy: strontium isotopes of a human deciduous tooth

Federico Lugli[a], Anna Cipriani[a,b], Julie Arnaud[c], Marta Arzarello[c], Carlo Peretto[c], Stefano Benazzi[d,e]

[a]Department of Chemical and Geological Sciences, University of Modena and Reggio Emilia, Via Campi 103, 41125 Modena, Italy
[b]Lamont-Doherty Earth Observatory, Columbia University, Palisades, New York 10964, USA
[c]Department of Humanities, Section of Prehistorical and Anthropological Sciences, University of Ferrara, C.so Ercole I d'Este 32, 44121 Ferrara, Italy
[d]Department of Cultural Heritage, University of Bologna, 48121 Ravenna, Italy
[e]Department of Human Evolution, Max Planck Institute for Evolutionary Anthropology, 04103 Leipzig, Germany

Strontium isotope ratios as proxy for mobility

In nature, strontium is a trace element commonly found in rocks of the Earth's crust. However, because one of its isotopes, namely ^{87}Sr, is continuously produced by radioactive decay of ^{87}Rb, with a half-life of 48.8 Ga, the Earth's rocks acquire different $^{87}Sr/^{86}Sr$ ratios in relation to their age and initial Rb abundance. Following erosion and weathering of the bedrock, Sr enters the ecosystem reaching plants and animals, mainly through water, and is fixed in the hydroxyapatite of teeth and bone tissue substituting calcium. As a result, the $^{87}Sr/^{86}Sr$ ratios in bone and teeth reflect the Sr isotopic ratio of the area where the tissue has formed, i.e. where the individual lived.

In the last decade, studies of enamel tissue have received much attention because enamel is a 96% mineralized tissue mainly composed by hydroxyapatite that is very resistant to diagenetic contamination and usually preserve the biogenic isotopic signature of the individuals better than bones (e.g. 1; for a complete review see 2). Moreover, given that enamel forms during childhood, its Sr isotopic composition should reflect the signature of the place where the individual has spent its youth, being a perfect target to unravel long-term human migration.

Primary teeth calcification

Primary teeth calcification (amelogenesis) begins in utero between the 13th and the 16th week of pregnancy and takes about 2-3 years to root completion. At birth, about 2/3 of the deciduous incisor enamel is formed (3). After birth, the first part of the tooth that reaches complete calcification is the crown; this process takes from about 2 months for the incisors to 11 months for the molars (3). Since strontium is fixed during the amelogenesis

calcification process, the Sr of the infant should derive from the food intake during this period, namely the fluids from the woman in utero and milk from breastfeeding. Thus, the $^{87}Sr/^{86}Sr$ ratio of the deciduous tooth enamel should reflect the $^{87}Sr/^{86}Sr$ of the woman during pregnancy and the first months of breastfeeding, which, in turns, reflect the living locality of the mother.

Isotopic composition of a deciduous tooth

To date it is not clear whether a deciduous tooth may retain chronological isotopic information. The early studies of Gulson and coworkers (4) on Pb isotopic composition of mother and child tissues showed that this element suffers large remobilization from old bone storage, causing a likely homogeneous composition of the child tissues because of the mother Pb buffering (4). However, compared to Sr, Pb has an extremely long residence time and its response to diet changes is thus quite slow. In their work on human hair, Font et al. (5) showed that Pb isotopes of hair (an incremental tissue, similar to enamel) are very slow in term of response to exposure changes, with occasionally even absent intra-individual variations after long-distance movement. On the contrary, the Sr isotope signal response to human movement is quite fast (monthly scale) and clear, even if it could take several months (up to 18) to reach the exact range of new local values (5).

No studies are currently present in literature about the Sr isotope intra-tooth variability of deciduous enamel. The work of Humphrey et al. (6) about Sr/Ca distributions in human deciduous teeth clearly shows time-related variations in term of Sr content after the beginning of the breastfeeding or the introduction of complementary food, suggesting that deciduous teeth can retain chronological information of diet changes. Sr isotopes were not analyzed in this study and it is not known if they are also variable. However, we could expect at least Sr isotope variations between the in-utero period and the subsequent

breastfeeding phase.

Even considering the enamel of permanent teeth the topic is highly debated. It is not clear if the Sr is fixed within the enamel during the secretory or the mineralization phase of the tooth formation. While some work states that the Sr isotope composition is averaged during the mineralization phase (7), others say that the greatest portion of Sr in enamel is fixed during the secretion stage (6). However, several authors (e.g. 8-11) have found significant sub-annual variations of the Sr isotopic ratio, in contrast to what observed by (7). Nevertheless, we cannot exclude that this is the result of species physiological differences. Nevertheless, the high correlation of the Sr isotope profiles of the inner enamel portion (nearby the EDJ) and the outer enamel surface found in (11), suggests that even after the last mineralization wave (outer enamel) the primary isotopic signal seems preserved.

Local isotopic signature

In isotope provenance study, the identification of the local isotopic signature can be challenging. In fact, several sources of Sr (e.g. weathered rocks, rainwater, rivers, underground stream) can contribute to the isotope composition of water and soil, which are the main reservoirs of bioavailable strontium for local animals. Rainwater can contribute from 10 to 50% of the Sr isotope local budget (references). The higher effect of rainwater to the local Sr is observed for coastal sites (8 and reference therein), where the local Sr ratio tends to 0.7092 (modern seawater). Accordingly, Italian rain Sr isotopic composition should be influenced by the seawater Sr and therefore this might influence the Sr isotopic composition of the bioavailable Sr towards $^{87}Sr/^{86}Sr$ values <0.7092.

Two strategies are commonly employed to determine the local $^{87}Sr/^{86}Sr$ baseline of the area of interest. The first one uses the Sr isotopic composition of archaeological animal teeth of species that, from an ecological point of view, are known to live locally without

migrating. Rodents, in particular, show a very narrow home range, generally lower than 1-2 km (12), being suitable for the determination of the local $^{87}Sr/^{86}Sr$ ratio. The second approach takes advantage of the availability of local modern animals, water and/or plants that thrive in the area of interest (2). Their Sr isotopic composition variability should reflect that one of the bioavailable Sr of the area. Moreover, the comparison with macro-mammals with a known wider home range (e.g. *Bison schoetensacki* and *Stephanorhinus hundsheimensis*) allows to unravel possible routes taken by the local hominin group (13).

Bioavailable strontium in central-southern Italy

Central and southern Italy are mainly characterized by sedimentary rocks dated between the Cretaceous and the Holocene. In Tuscany, some Palaeozoic basement rocks are exposed, but both Tuscany and Latium are dominated by volcanic rocks of the Cenozoic era (14). The current lack of a broad dataset of the isotopic composition of the Italian bioavailable strontium is a great limitation for provenance studies. While the $^{87}Sr/^{86}Sr$ ratio of limestone and other sedimentary rocks can be inferred from the marine seawater curve of McArthur (15), for metamorphic and magmatic rocks this is obviously not possible. Moreover, the bioavailable Sr depends also on other factors rather than solely on bedrock type as Sr is transported from further away in water and rainwater. However, based on what we can infer from the McArthur curve, central-southern Italy $^{87}Sr/^{86}Sr$ ratios of outcropping rocks should lie somewhere between the 0.707 ratio of the oldest Cretaceous sedimentary sequences and the modern seawater signal (0.7092). Higher values can be due to the presence of volcanic rocks from Latium (i.e. Colli Albani >0.710) and Campania (i.e. Roccamonfina 0.709 – 0.710).

Geological setting of Isernia La Pineta

Isernia La Pineta is located in the Apennines in the upper part of the Volturno River basin at 457 m above sea level. The archeological site was discovered in the quaternary fluvial-lacustrine sedimentary sequence that filled the Isernia basin, a tectonic depression associated with the Carpino-Le Piane Fault System still active today (CLPBFS; 16,17). During the Middle Pleistocene, the area was characterized by the presence of a major active volcano, Roccamonfina, which has produced many Plinian and ultra Plinian eruptions concurrently to the deposition of the fluvial-lacustrine succession. The stratigraphic sequence of the Isernia depression is characterized by two main morpho-lithostratigraphic units: Main Infill Unit and Valley Floor Unit. The Main Infill Unit was deposited in the Early/Middle Pleistocene and is composed by about 60 m thick gravel, silt and clay deposits of alluvial fan, fluvial and lacustrine environments, with several intercalated tephra layers presumably originated from the eruptive activity of the Roccamonfina Volcano. At the top of the Main Infill Units, depositional terraces (I order terraces) cut by erosional surfaces (II order terraces) have been recognized. The valley Floor Units consists of 5 to 15 m thick polygenetic gravels, with minor alluvial fanglomerates and scree deposits, in erosional contact with the Main Infill Unit and associated with depositional terraces of III and IV order (18-20).

Archeological context of the Isernia La Pineta site

The open-air site of Isernia la Pineta is characterized by an abundant lithic industry and faunal remains distributed in four archaeosurfaces and two sectors (3c, 3a, 3s10 in sect. I, 3a in sect. II). $^{40}Ar/^{39}Ar$ dating on sanidine crystals from several stratigraphic units recorded an age of 586 ± 1 ka (20).

The faunal assemblage, attributed to the Middle Galerian and consistent with the Ar/Ar

dating, is composed of *Palaeoloxodon antiquus*, *Stephanorhinus hundsheimensis*, *Hippopotamus* cf. *antiquus*, *Premegaceros solilhacus*, *Cervus elaphus* cf. *acoronatus*, *Bison schoetensacki*, *Hemitragus* cf. *bonali*, *Ursus deningeri* and *Panthera pardus*. Numerous cutmarks and intentional fresh bones fractures were found on herbivores demonstrating an intensive exploitation of carcasses (21,22). The presence of small mammals and particularly of *Sorex* aff. *runtonensis*, *Pliomys episcopalis*, *Microtus* (*Terricola*) *arvalidens* and *Arvicola mosbachensis*, allow to collocate the faunal assemblage to the Early Toringian and indicate that the climate was probably more arid and cooler than today and the environment was an arboreal steppe (20).

The lithic assemblage is mainly characterized by the exploitation of local flint slabs and limestone cobbles mainly by direct percussion by hard stone hummer and, when the raw material was of bad quality, by bipolar percussion on anvil. The reduction sequences are mostly short due to the small dimensions of the exploited raw material, and the cores were exploited by unipolar, multidirectional and centripetal (cf. discoid) methods. The flakes obtained are not standard in shape but are mostly thick, short and often overflowing. Cutting edges were modified for the production of sidescrapers and denticulates. The exploitation of local limestone was commonly for debitage and rarely for shaping. The debitage reduction sequences consist in the exploitation of one to three striking platforms by unipolar methods. In some cases, a functional edge was created by shaping on flat cobbles (23,24).

The human deciduous incisor (here after called IS42) was recovered from the level 3coll of Isernia La Pineta (Molise, Italy) (20). IS42 belonged to an individual of 5-7 years, possibly corresponding to the age-at-death considering the rather low degree of root resorption (Res ½, 17). The poor reference collection of deciduous teeth from the Middle Pleistocene chronological range precludes any definitive taxonomical consideration. Therefore, given the high morphological variability of Middle Pleistocene hominins, the tooth has been

attributed to an undetermined species of the genus *Homo* (i.e. *Homo* sp., 20).

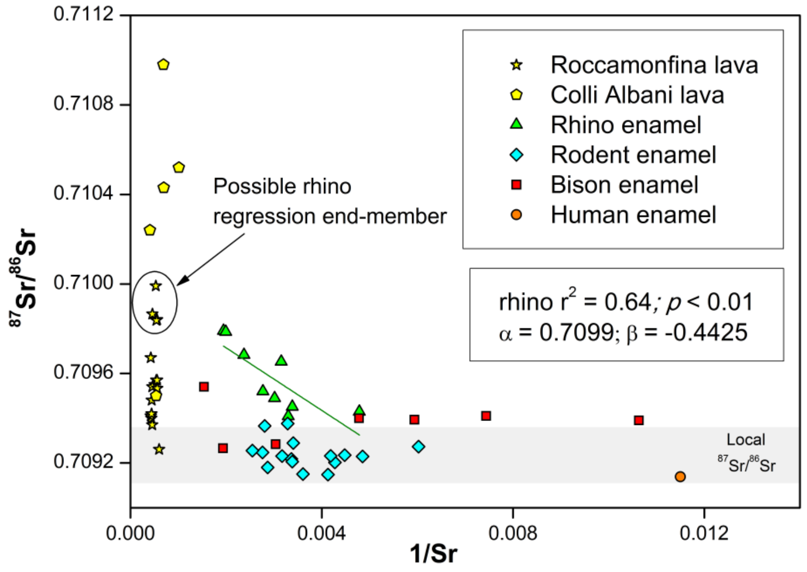

Figure S1. $^{87}Sr/^{86}Sr$ vs.1/Sr (ppm) . The grey area is the 2σ of the rodent specimens, representing the local bioavailable Sr range. The best-fit enriched end-member for the rhino correlation are the Roccamonfina lavas (Conticelli et al., 2009; Hawkesworth & Vollmer, 1979).

Table S1. Summary of dissolution MC–ICPMS results of fauna enamel samples from Isernia La Pineta site.

Species	Sample	Archaeological layer	$^{87}Sr/^{86}Sr$	Sr (ppm)
Microtinae indet	IS–R–1Sr	3c	0.70922	297
	IS–R–2Sr	3c	0.70915	277
	IS–R–3Sr	3c	0.70925	362
	IS–R–4Sr	3c	0.70926	392
	IS–R–5Sr	3c	0.70920	234
	IS–R–6Sr	3c	0.70927	166
	IS–R–7Sr	3s6-9	0.70921	295
	IS–R–8Sr	3s8-9	0.70923	206
	IS–R–9Sr	3s8-9	0.70924	223
	IS–R–10Sr	3s8-9	0.70915	242
	IS–R–11Sr	3s6-9	0.70923	239
	IS–R–12Sr	3s6-9	0.70923	315
	IS–R–13Sr	3s10	0.70937	356
	IS–R–14Sr	3s1-5	0.70918	348
	IS–R–15Sr	3s1-5	0.70929	294
	IS–R–16Sr	3s1-5	0.70938	304
Bison schoetensacki	IS-BS-17Sr	3s6-9	0.70941	134
	IS-BS-18Sr	3c	0.70940	209
	IS-BS-19Sr	3s6-9	0.70939	94
	IS-BS-20Sr	3s10	0.70954	649
	IS-BS-21Sr	3s10	0.70928	329
	IS-BS-22Sr	3c	0.70927	517
	IS-BS-23Sr	3c	0.70939	168
Stephanorhinus hundsheimensis	IS-RH-24Sr	3c	0.70965	317
	IS-RH-25Sr*	3c	0.70979	514
	IS-RH-26Sr*	3c	0.70979	501
	IS-RH-27Sr	3s10	0.70945	296
	IS-RH-28Sr	3s10	0.70941	303
	IS-RH-29Sr	3s10	0.70968	421
	IS-RH-30Sr	3s10	0.70952	361
	IS-RH-31Sr	3s10	0.70949	332
	IS-RH-32Sr	3s10	0.70943	209

All $^{87}Sr/^{86}Sr$ 2SE (in-run error) are < 0.00001. Sr concentration was obtained with a quadrupole ICPMS.
* Different portions of the same tooth.

**Table S2.** Location and strontium isotope ratios of modern plant samples				
**Location**	**Coordinates**	**$^{87}Sr/^{86}Sr$ modern plants**	**Bedrock**	**Distance from Isernia (km)**
Monteroduni	41°32'32.4" N 14°10'08.0" E	0.7085	Paleocene calcarenite	8
Trignete	41°32'47.4" N 14°15'31.7" E	0.7086	Langhian calcarenite	5
San Massimo	41°29'30.4" N 14°24'40.3" E	0.7096	Messinian sandstone	18
S. Stefano	41°36'58.7" N 14°36'49.0" E	0.7084	Messinian sandstone	30
Frosolone	41°37'56.9" N 14°23'10.1" E	0.7087	Eocene calcarenite	13
Castropignano	41°37'22.2" N 14°34'52.6" E	0.7088	Oligocene claystone	29
Vastogirardi	41°44'13.6" N 14°14'00.1" E	0.7083	Messinian sandstone	16
Aliano	41°23'44.1" N 14°13'20.6" E	0.7087	Trias dolostone	23
Bottazzella	41°32'21.5" N 14°03'19.8" E	0.7085	Paleocene calcarenite	16
Scapoli	41°36'47.3" N 14°03'26.3" E	0.7090 (0.7088)*	Messinian marl	15
Collalto	41°36'23.9" N 14°02'15.9" E	0.7096	Messinian marl	17
Letino	41°27'15.6" N 14°15'07.0" E	0.7087	Giuras limestone	17
Isernia La Pineta	41°35'23.4" N 14°14'41.9" E	0.7090	Pleistocene fluvio-lacustrine deposit	0
Roccamonfina**	41°18'59" N 13°58'47" E	0.7093	Middle Pleistocene leucite	40
Roccamonfina**	41°21'35" N 12°57'33" E	0.7100	Middle Pleistocene leucite	40

Propagated 2σ errors for all the measured ratios are ~0.00001.
*Snail sample.
**Leucite rock values from Conticelli et al. 2009 with reported the most and the least radiogenic ratios.

**Table S3.** Variability of the bioavailable strontium of the area surrounding Isernia La Pineta.				
**Distance from Isernia La Pineta**	**Mean $^{87}Sr/^{86}Sr$**	**2σ $^{87}Sr/^{86}Sr$**	**Max $^{87}Sr/^{86}Sr$**	**Min $^{87}Sr/^{86}Sr$**
< 15 km	0.7087	0.0005	0.7090	0.7085
> 15 km	0.7088	0.0011	0.7096	0.7083
Roccamonfina* (40 km SW)	0.7097	0.0006	0.7100	0.7093

*data from Conticelli et al., 2009.

Supplementary Information References

1. Lee-Thorp JA, Sponheimer M (2003) Three case studies used to reassess the reliability of fossil bone and enamel isotope signals for paleodietary studies. *J Anthropol Archaeol* 22:208–216.
2. Bentley RA (2006) Strontium isotopes from the earth to the archaeological skeleton: A review. *J Archaeol Method Theory* 13:135–187.
3. Nelson SJ, Ash MM (2010) Wheeler's Dental Anatomy, Physiology, and Occlusion, 9th Edition (Saunders/Elsevier).
4. Gulson BL, Pounds JG, Mushak P, Thomas BJ, Gray B, Korsch MJ (1999) Estimation of cumulative lead releases (lead flux) from the maternal skeleton during pregnancy and lactation. *Journal of Lab Clin Med* 134:631-640.
5. Font L, van der Peijl G, van Wetten I, Vroon P, van der Wagt B, Davies G (2012) Strontium and lead isotope ratios in human hair: investigating a potential tool for determining recent human geographical movements. *J Anal At Spectrom* 27:719-732.
6. Humphrey LT, Dean CM, Jeffries TE, Penn M (2008) Unlocking evidence of early diet from tooth enamel. *PNAS* 105:6834-6839.
7. Montgomery J, Evans JA, Horstwood MSA (2010) Evidence for long-term averaging of strontium in bovine enamel using TIMS and LA-MC-ICP-MS strontium isotope intra-molar profiles. *Environmental Archaeology* 15:32-42.
8. Bentley RA, Knipper C (2005) Transhumance at the early Neolithic settlement at Vaihingen (Germany). *Antiquity* 79 (306). antiquity.ac.uk/projgall/bentley306/
9. Balter V, Telouk P, Reynard B, Braga J, Thackeray F, Albarède F (2008) Analysis of coupled Sr/Ca and 87Sr/86Sr variations in enamel using laser-ablation tandem quadrupole-multicollector ICPMS. *Geochim Cosmochim Acta* 72:3980-3990.
10. Reitmaier T, Doppler T, Pike AW, Deschler-Erb S, Hajdas I, Walser C, Gerling C (2017) Alpine cattle management during the Bronze Age at Ramosch-Mottata, Switzerland. *Quat Int*. doi.org/10.1016/j.quaint.2017.02.007.
11. Lugli F, Cipriani A, Peretto C, Mazzucchelli M, Brunelli D (2017) In situ high spatial resolution 87Sr/86Sr ratio determination of two Middle Pleistocene (ca 580ka) *Stephanorhinus hundsheimensis* teeth by LA–MC–ICP–MS. *Int J Mass Spectrom* 412:38-48.
12. Clapperton B. K. (2006) A review of the current knowledge of rodent behaviour in relation to control devices. *Science for Conservation* 263 (Science & Technical Pub.).

13. Britton K, Grimes V, Niven L, Steele TE, McPherron S, Soressi M, Kelly TE, Jaubert J, Hublin JJ, Richards MP (2011) Strontium isotope evidence for migration in late Pleistocene Rangifer: implications for Neanderthal hunting strategies at the Middle Palaeolithic site of Jonzac, France. *J Hum Evol* 61:176-185.
14. Lee-Thorp JA, Sponheimer M (2003) Three case studies used to reassess the reliability of fossil bone and enamel isotope signals for paleodietary studies. *J Anthropol Archaeol* 22:208–216.
15. McArthur JM, Howarth RJ, Bailey TR (2001) Strontium isotope stratigraphy: LOWESS version 3: best fit to the marine Sr-isotope curve for 0–509 Ma and accompanying look-up table for deriving numerical age. *J of Geology* 109:155–170.
16. Di Bucci D, Naso G, Corrado S, Villa IM (2005) Growth, interaction and seismogenic potential of coupled active normal faults (Isernia Basin, central-southern Italy). *Terra Nov.* 17:44–55.
17. Macchiavelli C, Mazzoli S, Megna A, Saggese F, Santini S, Vitale S (2012) Applying the Multiple Inverse Method to the analysis of earthquake focal mechanism data: New insights into the active stress field of Italy and surrounding regions. *Tectonophysics* 580:124–149. doi:10.1016/j.tecto.2012.09.007
18. Brancaccio L, Di Crescenzo G, Rosskopf C, Santangelo N, Scarciglia F (2000) Carta Geologica Dei Depositi Quaternari e Carta Geomorfologica Dell'Alta Valle Del Fiume Volturno (Molise, Italia Meridionale). Note Illustrative. *Ital J Quat Sci* 13:81–94.
19. Coltorti M, Féraud G, Marzoli A, Ton-That T, Voinchet P, Bahain J-J, Minelli A, Thun Hohenstein U, Peretto C (2005) New 40Ar/39Ar, stratigraphic and palaeoclimatic data on the Isernia la Pineta Lower Palaeolithic site, Molise, Italy. *Quat Int* 113:11–22.
20. Peretto C, Arnaud J, Moggi-Cecchi J, Manzi G, Nomade S, Pereira A, Falguères C, Bahain JJ, Grimaud-Hervé D, Berto C, Sala B, Lembo G, Muttillo B, Gallotti R, Thun Hohenstein U, Vaccaro C, Coltorti M, Arzarello M (2015) A human deciduous tooth and new 40Ar/39Ar dating results from the Middle Pleistocene archaeological site of Isernia La Pineta, southern Italy. *PLoS One* 10:1–19.
21. Alhaique F, Bisconti M, Bietti A, Castiglioni E, Cilli C, Fasani L, Giacobini G, Grifoni R, Guerreschi A, Iacopini A, Malerba G, Peretto C, Recchi A, Rocci Riss A., Ronchitelli A, Rottoli M, Thun Hohenstein U, Tozzi C, Visentini P, Wilkens B (2004) Animal resources and subsistence strategies. *Collegium Antropologicum* 28:23–40.

22. Thun Hohenstein U, Di Nucci A, Moigne AM (2009) Mode de vie à Isernia La Pineta (Molise, Italie). Stratégie d'exploitation du Bison schoetensacki par les groupes humains au Paléolithique inférieur. *L'Anthropologie* 113:96–110. doi:10.1016/j.anthro.2009.01.009
23. Gallotti R, Peretto C (2015) The Lower/early Middle Pleistocene small débitage productions in Western Europe: New data from Isernia La Pineta t.3c (Upper Volturno Basin, Italy). *Quat Int* 357:264–281.
24. Parfitt SA, Barendregt RW, Breda M, Candy I, Collins MJ, Coope GR, Durbidge P, Field MH, Lee JR, Lister AM, Mutch R, Penkman KEH, Preece RC, Rose J, Stringer CB, Symmons R, Whittaker JE, Wymer JJ and Stuart AJ (2005) The earliest record of human activity in northern Europe. *Nature* 438:1008–1012.
25. Moorrees CFA (1963) Formation and resorption of three deciduous teeth in children. *Am J Phys Anthr* 21:205–213.
